# ﻿Phylogenetic conservation and diversification of 18S rDNA loci in leaf-cutting ants (Hymenoptera, Formicidae): insights from molecular validation and chromosomal mapping using FISH

**DOI:** 10.3897/compcytogen.20.162616

**Published:** 2026-01-05

**Authors:** Danon Clemes Cardoso, Maykon Passos Cristiano

**Affiliations:** 1 Departamento de Biodiversidade Evolução e Meio Ambiente, Universidade Federal de Ouro Preto, Ouro Preto, MG, Brazil Universidade Federal de Ouro Preto Ouro Preto Brazil

**Keywords:** Comparative phylogenetics, FISH-probe, leafcutting ants, rDNA, 18S rDNA, 45S ribosomal DNA

## Abstract

Ribosomal DNA (rDNA) clusters are important cytogenetic markers that can inform both taxonomic delimitation and chromosomal evolution in ants. In this study, we molecularly characterize and validate the widely used 18S rDNA probe applied in cytogenetic studies of Hymenoptera and provide new FISH-based chromosomal data for two previously unstudied leaf-cutting ant species (*Acromyrmex ambiguus* (Emery, 1888) and *Ac. crassispinus* (Forel, 1909)). While the general distribution of 45S rDNA loci in leafcutting is relatively well documented (copy number and site), we expand the comparative framework by testing the phylogenetic structure of rDNA positioning across genera. Our results confirm the conserved number of rDNA loci per species but reveal lineage-specific variation in chromosomal location, including both subterminal and pericentromeric arrangements. Phylogenetic signal analyses suggest non-random patterns consistent with evolutionary constraints in locus positioning. Together, our findings refine current cytogenetic models for leafcutting ants and demonstrate the utility of rDNA as a cytotaxonomic character and evolutionary marker for assessing chromosomal diversification.

## ﻿Introduction

In the early stages of molecular cytogenetics, chromosomal characterization relied heavily on base-specific fluorochromes. Distamycin A combined with 4’,6-diamidino-2-phenylindole (DAPI) enabled the detection of AT-rich regions, while chromomycin A3 (CMA₃) revealed GC-rich domains (Schweizer 1980; Cristiano et al. 2013; Cardoso et al. 2014; Barros et al. 2016; Teixeira et al. 2017; Cardoso et al. 2018; Teixeira et al. 2021a; Cardoso et al. 2022). The advent of fluorescence in situ hybridization (FISH) profoundly transformed cytogenetic research by enabling precise localization of DNA sequences on chromosomes through hybridization of fluorescently labeled probes (Rudkin and Stollar 1977). Earlier applications relied on radioactive probes (Pardue and Gall 1969), but the refinement of non-radioactive labeling has made FISH a cornerstone of modern chromosome biology.

FISH was first applied to ants in the late 1990s (Hirai et al. 1994; Lorite et al. 1997), and after a period of relative dormancy, it has become a standard tool in ant cytogenetics, particularly for mapping repetitive genomic elements such as ribosomal genes (e.g. Mariano et al. 2008; Santos et al. 2010; Teixeira et al. 2017, 2021a; Micolino et al. 2019b), microsatellite arrays (Micolino et al. 2019a, 2021), and telomeric motifs (Pereira et al. 2018; de Castro et al. 2020; Micolino et al. 2020). These repetitive elements define chromosomal compartments—such as telomeres, centromeres, and nucleolar organizing regions (NORs)—which are essential to genome organization and chromosomal behavior during cell division. Understanding their distribution and evolutionary dynamics is critical to deciphering the mechanisms of chromosomal evolution. FISH probes targeting rDNA were initially obtained from bacterial clones (Hirai et al. 1994), but are now routinely generated through PCR from genomic DNA using fluorescently labeled nucleotides. Despite widespread use, many primers employed for rDNA probe amplification have not been molecularly characterized, leaving uncertainties regarding their specificity and hybridization properties. Given that FISH relies on precise nucleotide complementarity, characterizing the molecular composition of these probes is crucial to standardize protocols and ensure reproducibility across studies.

Among these compartments, ribosomal DNA (rDNA) families—particularly the 45S rDNA cluster encompassing the 18S, 5.8S, and 28S rRNA genes—have emerged as highly informative cytogenetic markers. The 18S rDNA, part of the small subunit of eukaryotic ribosomes, is not only structurally conserved but also shows lineage-specific patterns of chromosomal positioning across taxa (Gokhman and Kuznetsova 2024). In ants, variation in rDNA site number and chromosomal location has been systematically documented across taxa, revealing both conserved patterns—such as a single rDNA-bearing chromosome pair—and notable positional differences among lineages. These comparative cytogenetic surveys highlight the frequent occurrence of rDNA sites in pericentromeric and interstitial regions, particularly within Myrmicinae, and provide a basis for inferring potential mechanisms such as centromere repositioning and pericentric inversions (Teixeira et al. 2021b; Damasceno et al. 2024). While these studies do not explicitly address evolutionary processes, their detailed chromosomal mappings offer a framework for subsequent evolutionary interpretations. Recent reviews have emphasized that the presence of a single 45S and 5S rDNA cluster in the haploid set is likely ancestral across Insecta, but extensive lineage-specific variation in number and chromosomal position exists, particularly among Hymenoptera (Gokhman and Kuznetsova 2024). This variability likely results from a combination of ectopic recombination and chromosomal fission events. Indeed, surveys across Hymenoptera have further emphasized the evolutionary relevance of rDNA loci, revealing a conserved pattern of single-site organization per haploid genome and a lineage-specific tendency for proximal chromosomal localization, particularly in groups with high karyotypic diversity such as ants and wasps (Menezes et al. 2021).

Moreover, ribosomal DNA loci have been recognized as potential hotspots for recombination due to their tandemly repeated structure and high transcriptional activity. The repetitive nature of rDNA arrays can promote unequal crossing over, gene conversion, or ectopic recombination events—processes that may lead to chromosomal rearrangements, such as translocations or inversions (Cazaux et al. 2011; McStay 2016; Li et al. 2022). While such dynamics may contribute to genomic instability, they can also drive karyotypic innovation and speciation. The positioning of rDNA clusters—whether terminal, pericentromeric, or interstitial—may therefore reflect not only structural constraints but also evolutionary pressures to mitigate or harness these recombinogenic properties. Investigating the chromosomal context of rDNA loci in ants offers a valuable opportunity to explore how genome architecture evolves under the dual forces of stability and plasticity.

Current phylogenetic frameworks have enhanced our understanding of chromosome number evolution in ants, revealing that variation in haploid chromosome number is shaped primarily by recurrent dysploidy events—both ascending (fissions) and descending (fusions)—and, to a lesser extent, by rare shifts in ploidy across some lineages (Cardoso and Cristiano 2025). Their ancestral state reconstruction supports an ancestral haploid number of n = 11 and highlights a trend toward karyotype diversification through chromosomal fragmentation and genome size reduction. These findings offer critical context for interpreting cytogenetic markers such as rDNA loci, which may also shift positionally across lineages experiencing chromosomal restructuring. In this context, we molecularly characterize a widely used 18S rDNA probe applied in FISH analyses of Neotropical Hymenoptera. We validate its specificity through sequencing and confirm its applicability across previously analyzed ant species. Additionally, we extend its use to *Acromyrmex ambiguus* (Emery, 1888) and *Acromyrmex crassispinus* (Forel, 1909), providing the first chromosomal mapping of 45S rDNA loci in these species. By integrating these new cytogenetic data with a genus-level phylogenetic framework, we investigate how the chromosomal positioning of rDNA sites varies among leaf-cutting ant genera and whether such variation is associated with differences in chromosomal morphology and lineage divergence. This combined approach enables a more comprehensive understanding of rDNA compartmental dynamics and their evolutionary implications within this ecologically specialized ant group.

This study aims to molecularly characterize and validate a routinely employed 18S rDNA probe in Neotropical Hymenoptera, with particular focus on its application in physical mapping of 45S rDNA clusters in leaf-cutting ants (genera *Acromyrmex* Mayr, 1865, *Amoimyrmex*Cristiano et al., 2020, and *Atta* Fabricius, 1804) (Cristiano et al. 2020), which represent an ecologically specialized group within the subtribe Attina (Myrmicinae: Attini). By integrating molecular sequencing, FISH-based chromosomal mapping, and comparative phylogenetic analysis, we examine the evolutionary dynamics of rDNA loci within these taxa. Specifically, we ask: (i) Is the chromosomal location of 18S rDNA conserved among leaf-cutting ants, or does it exhibit lineage-specific variability? (ii) Do patterns of rDNA site positioning exhibit phylogenetic signal across leaf-cutting ant genera? (iii) Can rDNA loci serve as reliable cytogenetic markers for inferring the evolution of chromosomal compartments—particularly telomeres, centromeres, and nucleolus organizing regions (NORs)—within this group of fungus-farming ants?

## ﻿Material and methods

### ﻿Biological samples and chromosome preparation

Colonies of *Acromyrmex crassispinus* were sampled in Ouro Preto, Minas Gerais, Brazil (20°17'15"S, 43°30'29"W), while *Acromyrmex ambiguus* was collected in Ilha Comprida, São Paulo, Brazil (24°44'28"S, 47°32'24"W). The species *Amoimyrmex bruchi* (Forel, 1912) and *Amoimyrmex silvestrii* (Emery, 1905) were sampled in Argentina (see Cristiano et al. 2020 for detailed locations). After collection in Brazil, colony fragments—including workers, queens, and brood (larvae and pupae)—were transported to the laboratory at the Universidade Federal de Ouro Preto (UFOP), Minas Gerais, Brazil, and maintained under laboratory conditions as described by Cardoso et al. (2011). In addition, workers from one colony of *Melipona quadrifasciata* (Lepeletier, 1836) were obtained from Viçosa, Minas Gerais. Slides of *Am. bruchi* and *Am. silvestrii* were prepared in Argentina.

Metaphase chromosomes of ants were obtained from the brain ganglia of pre-pupal larvae following the method of Imai et al. (1988), with time modifications as described by Cardoso et al. (2017). The larvae’s brain ganglia were dissected in a hypotonic colchicine solution (0.005% w/v colchicine in 1% sodium citrate solution) under a stereoscopic microscope. For each species analyzed, we examined at least 5 individuals and a minimum of 15 high-quality metaphase plates per species. The diploid chromosome number was determined based on well-spread metaphases and confirmed across multiple individuals.

### ﻿DNA extraction, polymerase chain reaction (PCR) and sequencing

Genomic DNA was isolated from the ant *Mycetophylax morschi* Emery, 1888 and the stingless bee *Melipona quadrifasciata* using proteinase K digestion followed by a standard CTAB protocol (Sambrook and Russell 2001). Fragments of the 18S rDNA gene were then amplified by PCR using the primers 18SF (5’-GTCATATGCTTGTCTCAAAGA-3’) and 18SR (3’-TCTAATTTTTTCAAAGTAAACGC-5’)^1^, to produce the probes used in FISH experiments. The source of the primers is controversial and is discussed further below. DNA amplification was conducted in reactions with a final volume of 25 μL, containing the following components: MgCl_2_ (2.5 mM), buffer (10x; Pro- mega), dNTPs (1 mM each), two primers (0.48 μM each), Taq polymerase (2 U of GoTaq® Flexi DNA Polymerase) and 1 μL of template DNA. PCR cycling conditions were as follows: initial denaturation for 3 min at 94 °C, then 35 cycles with 94 °C for 1 min denaturation, 55 °C for 1 min annealing, 72 °C for 1 min extension, and a final extension at 72 °C for 5 min. PCR products, which corresponded to a ~750-bp segment, were purified and sequenced using the same primers as for amplification in an ABI PRISM 3700 sequencing system.

### ﻿Fluorescence *in situ* hybridization

FISH experiments were performed as previously described by Kubat et al. (2008), with some modifications. The detailed procedures are as follows: the chromosomal preparations were incubated in RNAse (10 μg/mL) for one hours at 37 °C. After we washed for five minutes in 2 × SSC, pH 7.0 (0.03 M sodium citrate and 0.3 M sodium chloride), and 0.005% pepsin was added for 10 min at room temperature. We washed again in 1 × PBS (1.36 M sodium chloride and 0.07 M disodium phosphate) for five minutes followed by a time-out in 10% formaldehyde for ten minutes at room temperature, and again washed in 1 × PBS for five minutes. The chromosomal preparations on the slides were dehydrated in a series of alcoholic solutions for two minutes each (50, 70, and 100%, respectively) prior to denaturation by 70% formamide/2 × SSC at 75 °C on a heating plate for five minutes. Then, the spreads were dehydrated again in an alcoholic series of which the thermal shock in cold alcohol, and finally, the chromosomes were hybridized with previously amplified probe by mean of the mix (200 ng of labeled probe, 50% formamide, 2 × SSC, and 10% dextran sulfate 20 × SSC) and at 75 °C for ten minutes and reserved in a humid chamber at 37 °C overnight (i.e., the probe was hybridizing for at least 18 hours). The next day, the chromosomal preparations were washed in 2 × SSC and 1 × SSC for five minutes each, plus a further wash in 4 × SSC/Tween for five minutes. Then, slides were incubated in 3% NFDM 4 × SSC (i.e., a mix of distilled water, 20 × SSC, and powdered milk). A detection solution (3% NFDM 4 × SSC and anti-digoxigenin-rhodamine) was then added to each slide and kept in a moist chamber at 37 °C for one hour. Slides were assembled in antifade solution with DAPI (4’,6-diamidino-2-phenylindole) (Sigma-Aldrich®).

### Sequencing comparison

We sequenced the PCR-amplified probe of 18S rDNA for *Melipona quadrifasciata*, as this probe was first applied in bee studies but had never been verified. We also sequenced probes amplified based on the genome of *Mycetophylax morschi* for application in ant studies. All sequences obtained in this study were checked for quality using Geneious Prime software (Kearse et al. 2012), with nucleotides having a Phred score of < 20 trimmed from both sequence ends.

The nucleotide sequences (GenBank accession numbers: PX243334–PX243339) were aligned using the MUSCLE algorithm implemented in MEGA7 software (Kumar et al. 2016) and manually inspected. We then carried out a BLASTn analysis using default parameters (Zhang et al. 2000) and selected ant species for comparison. A total of six ant sequences (GenBank accession numbers: KJ860073, KJ860098, KJ860115, KJ860151, KJ860152, KJ860211) and one stingless bee sequence (*M. quinquefasciata* - AY773344) for the same gene fragments were retrieved from GenBank and aligned. To place our probes in a relative position, we compared our matrix of 18S rDNA fragments with the closest phylogenetically related species with a complete 18S sequence available in GenBank (*Aphelinus gossypii* Timberlake, 1924, Aphelininae, Aphelinidae, Chalcidoidea, Hymenoptera – GenBank accession number: AY216700) and subsequently with *Drosophila simulans* Sturtevant, 1919 (GenBank accession number: AY037174).

### Phylogenetic signal analysis of rDNA signal codes

To assess whether the chromosomal localization patterns of the 45S rDNA clusters exhibit phylogenetic structure among leaf-cutting ants, we tested the evolutionary signal of discrete cytogenetic states coded from fluorescence in situ hybridization (FISH) data. For this, each species was assigned a categorical variable (hereafter referred to as “signal code”) representing the position of the rDNA cluster, such as ST1-t (terminal on the first subtelocentric pair), ST1-sa (short arm of the first subtelocentric pair), M4-i (interstitial on the fourth metacentric pair), and M2-i (interstitial on the second metacentric pair).

We used a dated phylogenetic tree of fungus-farming ants based on multilocus molecular data from Cardoso and Cristiano (2025), pruned to include only the species for which rDNA signal codes were available. The tree, formatted in NEXUS, was processed in R v4.4.1 (R Core Team 2024) using the package ape (Paradis et al. 2004). The rDNA signal codes were treated as unordered discrete traits and analyzed with the function fitDiscrete from the Geiger package (Pennell et al. 2014), under an equal-rates (ER) transition model. This model estimates the evolutionary transition rates between discrete character states, allows inference of phylogenetic structuring of chromosomal features, and was further used to reconstruct ancestral character states for the rDNA positional codes. The estimated transition probabilities provided the most likely ancestral states at internal nodes of the pruned phylogeny.

To visualize the distribution of signal codes and reconstructed states across the phylogeny, we used the functions plotTree and tiplabels from the phytools package (Revell 2012). Tree tips were annotated with color-coded points corresponding to each rDNA positional code, while ancestral reconstructions were mapped onto internal nodes, enabling evolutionary interpretation of chromosomal localization patterns across leaf-cutting ants. Phylogenetic signal was assessed based on model likelihoods, AIC values, and the estimated transition matrix, and interpreted in light of chromosomal evolution hypotheses in Attina ants. To investigate the relationship between ribosomal gene (rDNA) signal types and evolutionary age, we performed point-biserial correlation tests between divergence time and the presence/absence of four rDNA signal categories: M2-i, M4-i, ST1-sa, and ST1-t across leaf-cutting ant species and genera.

## ﻿Results

### FISH-probes characterization

**Figure 1.**
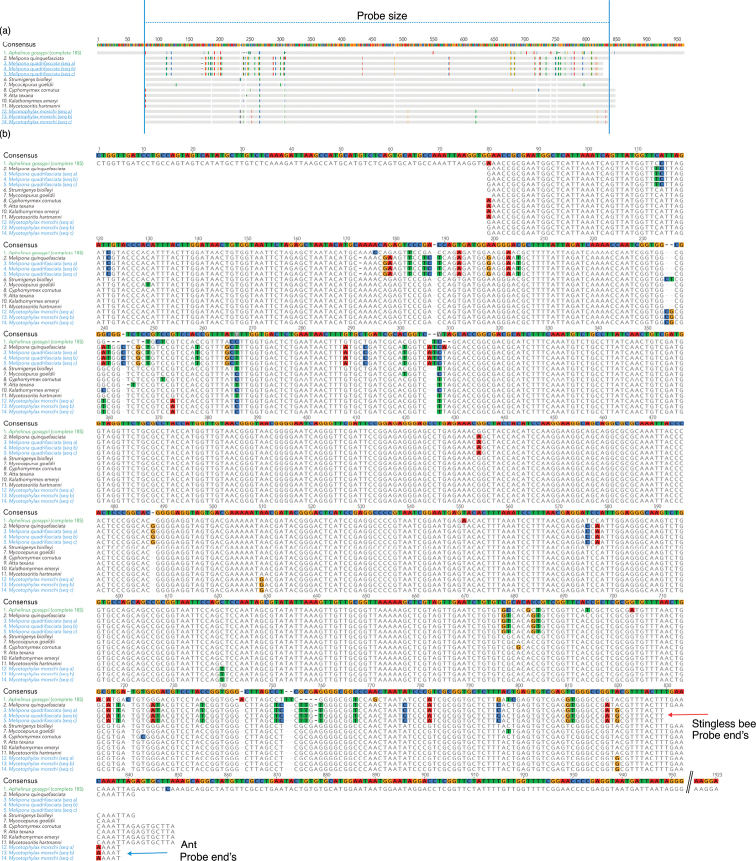
Sequence alignments were performed for 18S probes recovered from *Melipona quadrifasciata* and *Mycetophylax morschi*, along with the 18S rDNA gene from other ant and bee species. The alignment is based on the 18S rDNA gene of *Aphelinus gossypii* complete sequence, with the numbers corresponding to positions within this reference sequence **a** overview and length of the 18S probes at the beginning of the 18S rDNA gene **b** detailed alignment view. Blank spaces indicate gaps, while colored nucleotides highlight differences across species. Blue and red arrows mark the ends of each probe.

To validate the routine use of 18S rDNA probes in our cytogenetic analyses, we sequenced and characterized the probes previously applied in FISH experiments with ants. Although the 18S rDNA probe has been widely employed in karyotype studies of Hymenoptera (e.g., Teixeira et al. 2017; Micolino et al. 2019a; Cunha et al. 2023), its molecular composition had not yet been formally described. In this study, we provide the first molecular characterization of the ant-derived 18S rDNA probe used in our group’s FISH protocol. The probes amplified from ants yielded fragments ranging from 741 to 750 base pairs (bp), corresponding to the initial segment of the 18S gene. Sequence alignment revealed that the ant probe begins at position 80 of the complete *Aphelinus gossypii* 18S gene (Fig. 1). Despite slight interspecific differences, especially when compared to *Drosophila simulans*, the overall structure and conservation of the amplified region were consistent with expected eukaryotic rDNA organization.

**Figure 2.**
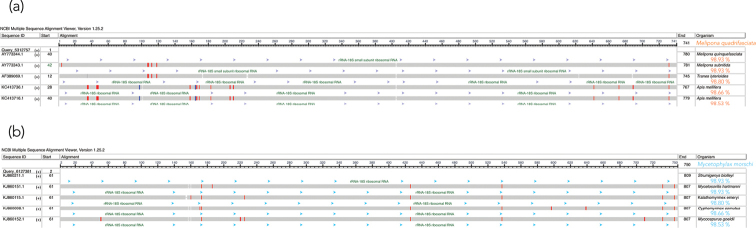
Graphic display of nucleotide BLAST (blastn) results for the 18S probe sequence **a** top five hits for *Melipona quadrifasciata***b** top five hits for *Mycetophylax morschi*. Red and blue bars highlight the differences. The percentage of similarity is shown above each organism’s name.

BLASTn analyses confirmed high specificity of the probes, with 99% sequence identity to 18S rDNA sequences from ants (Formicidae), validating their taxonomic origin and suitability for cytogenetic mapping (Fig. 2). Notably, although sequence variation exists between ants and stingless bees (*Melipona* spp.), especially across the probe extension (Fig. 1), cross-hybridization remains effective, supporting the robustness of the primers used for amplification. The base composition analysis revealed balanced proportions of A, T, C, and G across sequences, with a slight thymine enrichment in *Melipona* Illiger, 1806 probes (Table 2). Since thymine is the labeled base in the digoxigenin-11-dUTP system, slight PCR mix adjustments might be considered. However, the observed deviation (approximately 1%) is unlikely to compromise labeling efficiency.

Collectively, these results demonstrate that the ant-derived probe used in this study is highly conserved, effective for FISH detection of 45S rDNA loci, and now fully characterized for methodological reproducibility and future applications in cytogenetic studies of Formicidae.

### 18S rDNA characterization in Leaf-cutting ants

**Figure 3.**
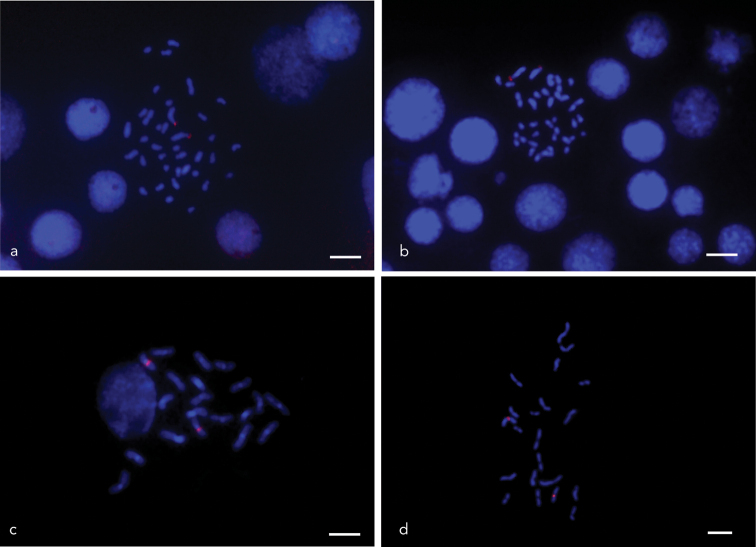
Fluorescence in situ hybridization (FISH) using an 18S rDNA probe on metaphase chromosomes of leaf-cutting ants **a***Acromyrmex ambiguus* and **b***Acromyrmex crassispinus*: 45S rDNA signals are reported here for the first time, located in the distal (telomeric) region of the short arm of the largest subtelocentric chromosome pair **c***Amoimyrmex bruchi* and **d***Amoimyrmex silvestrii*: 45S rDNA sites are consistently positioned in the proximal interstitial region of the second metacentric chromosome pair. Chromosomes were counterstained with DAPI. Scale bars: 5 µm.

Following the molecular validation of the 18S rDNA probe, we applied FISH to characterize the chromosomal localization of 45S rDNA in leaf-cutting ants of the genera *Acromyrmex* and *Amoimyrmex* (Fig. 3). In *Acromyrmex crassispinus* and *Ac. ambiguus*, the karyotype consists of 38 chromosomes, predominantly subtelocentric, with a single 45S rDNA cluster consistently located in the telomeric region of the short arm of the largest subtelocentric pair (ST1-t) (Fig. 3a, b). In *Amoimyrmex bruchi* and *Am. silvestrii*, the diploid number is 22, with chromosomes primarily metacentric. The 45S rDNA locus is interstitially located on the second metacentric pair (M2-i) (Fig. 3c, d). These findings are summarized in Table 1 and reflect the conserved number, but variable positioning, of rDNA loci across the studied taxa and those reported in the literature. The localization pattern observed in the present study for *Acromyrmex* spp. and *Amoimyrmex* spp. are consistent with previous findings (Table 1), suggesting a conserved chromosomal arrangement of rDNA within each genera.

**Table 1.** Summary of chromosomal localization patterns of 45S rDNA in leaf-cutting ants (Formicidae: Myrmicinae: Attini: Attina). The diploid number (2n), position of the rDNA signal on chromosomes, positional code used for phylogenetic analysis, and correspondin g references are indicated.

Species	2n	Location of rDNA signal	Code	Reference
***Acromyrmex ambiguus* (Emery, 1888)**	38	Terminal (1^st^ subtelocentric pair)	ST1-t	**This study**
***Acromyrmex ameliae* (De Souza et al. 2007)**	36	Terminal (1^st^ subtelocentric pair)	ST1-t	Barros et al. (2021)
***Acromyrmex aspersus* (Smith, 1858)**	38	Terminal (1^st^ subtelocentric pair)	ST1-t	Teixeira et al. (2017)
***Acromyrmex balzani* (Emery, 1980)**	38	Short-arm (1^st^ subtelocentric pair)	ST1-sa	Barros et al. (2024)
***Acromyrmex brunneus* (Forel, 1912)**	38	Terminal (1^st^ subtelocentric pair)	ST1-t	Barros et al. (2024)
***Acromyrmex coronatus* (Fabricius, 1804)**	38	Terminal (1^st^ subtelocentric pair)	ST1-t	Barros et al. (2016)
***Acromyrmex crassispinus* (Forel, 1909)**	38	Terminal (1^st^ subtelocentric pair)	ST1-t	**This study**
***Acromyrmex disciger* (Mayr, 1887)**	38	Terminal (1^st^ subtelocentric pair)	ST1-t	Barros et al. (2016)
***Acromyrmex octospinosus* (Reich, 1793)**	38	Terminal (1^st^ subtelocentric pair)	ST1-t	Teixeira et al. (2021b): as *Ac. echinatior*
***Acromyrmex laticeps* (Emery, 1905)**	38	Terminal (1^st^ subtelocentric pair)	ST1-t	Barros et al. (2024)
***Acromyrmex molestans* Santschi, 1925**	38	Terminal (1^st^ subtelocentric pair)	ST1-t	Teixeira et al. (2017)
***Acromyrmex niger* (Smith, 1858)**	38	Terminal (1^st^ subtelocentric pair)	ST1-t	Barros et al. (2016)
***Acromyrmex subterraneus* (Forel, 1893)**	38	Terminal (1^st^ subtelocentric pair)	ST1-t	Barros et al. (2024)
***Amoimyrmex bruchi* (Forel, 1912)**	22	Interstitial (2^nd^ metacentric pair)	M2-i	Micolino et al. (2021)/**this study**
***Amoimyrmex silvestrii* (Emery, 1905)**	22	Interstitial (2^nd^ metacentric pair)	M2-i	Micolino et al. (2021)/**this study**
***Amoimyrmex striatus* (Roger, 1863)**	22	Interstitial (2^nd^ metacentric pair)	M2-i	Teixeira et al. (2017)
***Atta bisphaerica* Forel, 1908**	22	Interstitial (4^th^ metacentric pair)	M4-i	Teixeira et al. (2017)
***Atta laevigata* (Smith, 1858)**	22	Interstitial (4^th^ metacentric pair)	M4-i	Teixeira et al. (2017)
***Atta robusta* Borgmeier, 1939**	22	Interstitial (4^th^ metacentric pair)	M4-i	Barros et al. (2015)
***Atta sexdens* (Linnaeus, 1758)**	22	Interstitial (4^th^ metacentric pair)	M4-i	Teixeira et al. (2017)

**Table 2.** Nucleotide proportion of nucleotides across 18S rDNA sequences studied here. A = adenine, T = thymine, C = cytosine, and G = guanine.

Species	T(U) (%)	C (%)	A (%)	G (%)	Total
*Aphelinus gossypii* - complete	24.9	22.9	24.9	27.2	1905
*Melipona quinquefasciata*	26.0	22.8	24.8	26.3	753
*Melipona quadrifasciata* (seq. a)	26.2	23.1	24.2	26.6	741
*Melipona quadrifasciata* (seq. b)	26.2	23.1	24.2	26.6	741
*Melipona quadrifasciata* (seq. c)	26.2	23.1	24.2	26.6	741
*Strumigenys biolleyi*	24.8	23.8	24.2	27.2	753
*Mycocepurus goeldii*	24.9	23.7	24.2	27.2	747
*Cyphomyrmex cornutus*	24.8	23.7	24.2	27.3	759
*Atta texana*	24.9	23.5	24.4	27.2	758
*Kalathomyrmex emeryi*	24.6	24.0	24.4	27.0	759
*Mycetosoritis hartmanni*	24.9	23.6	24.4	27.1	759
*Mycetophylax morschi* (seq. a)	24.8	23.7	24.1	27.3	750
*Mycetophylax morschi* (seq. b)	24.8	23.7	24.1	27.3	750
*Mycetophylax morschi* (seq. c)	24.8	23.7	24.1	27.3	750

### Phylogenetic structuring of rDNA signal codes

To explore the evolutionary patterns of rDNA localization in leaf-cutting ants, we compiled cytogenetic data from both our own FISH experiments and previously published studies available in the scientific literature and online databases (Table 1). The chromosomal localization of 18S rDNA in leaf-cutting ants showed a clear phylogenetic pattern. Most *Acromyrmex* species exhibited the ST1-t signal, corresponding to a terminal site on the first subtelocentric chromosome pair. In contrast, *Atta* species consistently presented the M4-i signal (interstitial site on the fourth metacentric pair). The M2-i code – representing an interstitial signal on the second metacentric pair – was observed in *Amoimyrmex bruchi*, *Am. silvestrii*, and *Am. striatus* (Roger, 1863), indicating a shared cytogenetic pattern distinct from other *Acromyrmex* species. An intermediate signal, ST1-sa (short arm of ST1), was reported in *Acromyrmex balzani* (Emery, 1980), possibly reflecting a transitional or derived arrangement.

**Figure 4.**
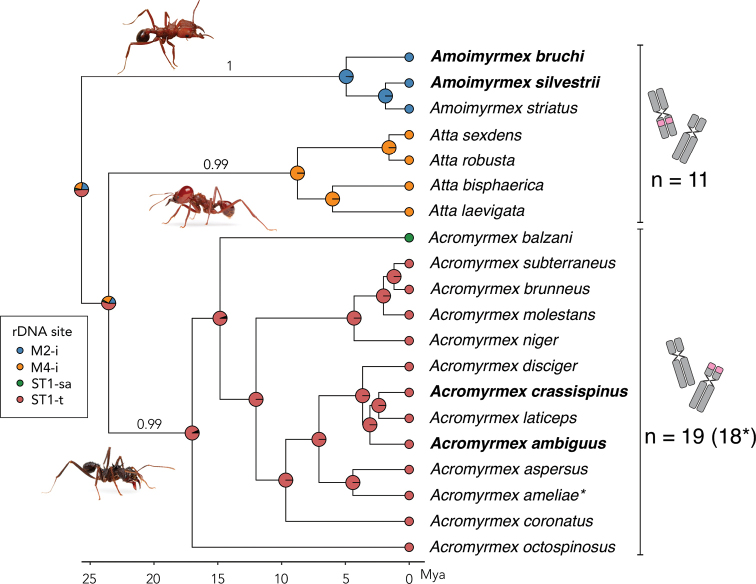
Phylogenetic distribution and ancestral state reconstruction of 45S rDNA chromosomal locations in leaf-cutting ants (Attina). The phylogeny, pruned from Cardoso and Cristiano (2025), includes all known species with cytogenetic data on 45S rDNA loci, combining newly generated results and published records (see Table 1). rDNA positions were determined via fluorescence in situ hybridization (FISH) and are shown at the tree tips as color-coded circles, with each color representing a specific chromosomal location: terminal on subtelocentric pair (ST1-t), short arm of subtelocentric pair (ST1-sa), interstitial on metacentric pair (M2-i), or pericentromeric on metacentric pair (M4-i). Species analyzed in this study are highlighted in bold. Pie charts at internal nodes represent ancestral state reconstructions under a maximum likelihood model, with segment sizes indicating the relative likelihood of each positional category. Dashes within pie charts indicate the boundaries between probability slices representing distinct positional states. In single-color charts (i.e., when a single state has near-100% probability), the dash simply marks the start and end point of the full 360° slice. The tree reveals strong phylogenetic structuring of rDNA localization, with genus-level clustering of chromosomal positions. The ant images represent typical morphology of each genus: *Acromyrmex*, *Amoimyrmex*, and *Atta*, and were obtained from ©Alex Wild’s photographic database, with permission.

Signal code distribution across the phylogeny was non-random, with closely related species sharing the same rDNA localization pattern (Fig. 4). This suggests evolutionary conservatism in ribosomal cluster positioning. Under an equal-rates (ER) model, transition rates between states were symmetrical (q ≈ 0.00685), with log-likelihood = –10.09 and AIC = 22.17.

The ancestral state reconstruction based on the discrete character representing the location of rDNA loci is depicted in Fig. 4. The pie charts at each node represent the proportional likelihoods of each ancestral state, inferred using maximum likelihood. The analysis supports the hypothesis that the observed patterns of rDNA localization are phylogenetically structured, with interstitial localization appearing as ancestral and retained state in some *Atta* and *Amoimyrmex* species, while the terminal localization is likely a derived state in *Acromyrmex*.

A significant positive correlation was found between the ST1-t signal and divergence time (r = 0.57; p = 0.009), supporting the hypothesis that this localization is a derived condition within *Acromyrmex*. The ST1-sa signal showed a moderate negative correlation (r = –0.39; p = 0.085), potentially representing an ancestral state. The M2-i and M4-i signals, restricted to specific genera (*Amoimyrmex* and *Atta*, respectively), showed no significant correlation with divergence time (p > 0.1), likely reflecting genus-specific chromosomal rearrangements. Together, these findings indicate that 18S rDNA positioning is phylogenetically informative and reflects both conserved and lineage-specific karyotypic evolution within leaf-cutting ants.

## ﻿Discussion

Following the molecular validation of the 18S rDNA probe, fluorescence in situ hybridization (FISH) was employed to determine the chromosomal localization of 45S rDNA clusters in leaf-cutting ants belonging to the genera *Acromyrmex* and *Amoimyrmex*. The probe, derived from ant genomic material, produced clear and consistent hybridization signals across all analyzed species, confirming its efficacy and specificity. In *Ac. crassispinus* and *Ac. ambiguus*, a single 18S rDNA locus was detected per haploid genome, consistently located at the telomeric region of the first subtelocentric chromosome pair. This pattern is consistent with previous observations in other *Acromyrmex* species (see Table 1), suggesting a conserved chromosomal arrangement of ribosomal DNA within the genus. Similarly, *Am. bruchi* and *Am. silvestrii* each exhibited a single hybridization signal, positioned interstitially on the second metacentric chromosome pair, in accordance with the configuration reported for congeneric taxa (Teixeira et al. 2017; Micolino et al. 2021). Despite interspecific variation in diploid chromosome number and morphology, the number of major 18S rDNA loci remained invariant (one locus per genome) highlighting the structural stability of rDNA-bearing chromosomes across these lineages and reinforcing the cytogenetic utility of this marker in comparative analyses within the Attina clade.

The conserved number of 18S rDNA loci across *Acromyrmex*, *Amoimyrmex* and *Atta*, despite marked differences in karyotype structure, suggests that rDNA-bearing chromosomes are subject to strong evolutionary constraints within leaf-cutting ants. However, the distinct patterns of chromosomal positioning observed among these genera indicate that, while the number of loci remains stable, their genomic location is more evolutionarily labile and potentially informative. The presence of a single rDNA-bearing chromosome pair was initially proposed as a plesiomorphic condition within Attina by Micolino et al. (2019b). Subsequently, a broader comparative survey across Formicidae confirmed that this condition, often involving interstitial localization of rDNA, is likely ancestral for ants (Teixeira et al. 2021b), and later to Insecta (Gokhman and Kuznetsova 2024).

Patterns of 18S rDNA localization in *Atta* and *Acromyrmex* were previously documented through FISH-based cytogenetic descriptions (Teixeira et al. 2017), but had not been assessed within a phylogenetic and evolutionary framework. Here, we empirically test for phylogenetic structuring in rDNA site localization and demonstrate that the observed patterns correspond closely with the evolutionary trajectories of *Acromyrmex*, *Amoimyrmex* and *Atta*. Our findings are consistent with the differential karyotypic dynamics reported for these genera, where *Acromyrmex* displays increased chromosomal variability compared to the more conserved karyotype of *Atta*. This contrast likely reflects shifts in chromosome evolution rates across lineages, as proposed by Cardoso and Cristiano (2024). These results support the hypothesis that, although the number of rDNA loci is evolutionarily constrained, their chromosomal positioning may have diversified in response to lineage-specific karyotypic changes within leaf-cutting ants.

The ancestral state reconstruction, visualized through pie charts at internal nodes of the phylogeny (Fig. 4), supports the hypothesis that distinct rDNA localization patterns are genus-specific and likely derived. For instance, the inferred ancestral state for *Acromyrmex* is strongly supported as ST1-t, while M4-i is recovered as the most probable ancestral condition for *Atta* species. These reconstructions further reinforce the interpretation that the observed rDNA positional states are not randomly distributed, but reflect lineage-specific chromosomal rearrangements that have been maintained through evolutionary time. Although deeper ancestral nodes are less resolved, no indication of polymorphism or homoplasy was observed among the genera sampled, suggesting that the evolutionary trajectory of rDNA loci is relatively stable once established within each lineage.

It is important to note that the phylogenetic analysis conducted here is based on a pruned tree including only species for which cytogenetic data on rDNA localization are available. While this approach ensures analytical focus, the exclusion of species with unknown rDNA patterns may, in theory, affect ancestral state reconstructions and estimates of phylogenetic signal. Future analyses incorporating full taxon sampling and coding unknown states explicitly may help refine these evolutionary inferences. Nevertheless, the strong genus-level clustering of rDNA positional states observed here supports the interpretation of these states as putative apomorphies, revealing clear phylogenetic structure in chromosomal compartmentalization across leaf-cutting ant lineages.

The predominance of the ST1-t signal in *Acromyrmex* species, coupled with its positive correlation with divergence time, supports the hypothesis that this arrangement represents an apomorphy of *Acromyrmex* and may be adaptive. Similarly, the interstitial M2-i configuration observed in *Amoimyrmex*, and the M4-i pattern characteristic of *Atta* are also phylogenetically restricted, likely reflecting genus-specific chromosomal rearrangements that have shaped karyotype structure within leaf-cutting ants (Cardoso and Cristiano 2024). Notably, *Acromyrmex* and *Atta* possess subtelocentric chromosomes, whereas *Amoimyrmex* does not. This suggests that the interstitial location of rDNA loci represents a retained (plesiomorphic) condition, while the terminal positioning on subtelocentric chromosomes constitutes a derived arrangement. These findings highlight the dual role of rDNA loci as both conserved markers for comparative cytogenetics and dynamic genomic elements that capture lineage-specific evolutionary trajectories. The non-random distribution of signal types across the phylogeny further highlights the utility of rDNA site localization as a cytotaxonomic character, providing enhanced resolution for reconstructing chromosomal evolution and diversification within fungus-growing ants. More broadly, our results suggest that the distribution of rDNA sites across Formicidae is shaped by both phylogenetic history and chromosomal rearrangements, expanding upon previous large-scale cytogenetic surveys that have mapped rDNA loci across multiple subfamilies (Teixeira et al. 2021b; Damasceno et al. 2024).

In all studied *Acromyrmex* species, including *Ac. ambiguus* and *Ac. crassispinus* reported here, the 45S rDNA is consistently located in the terminal region of the largest subtelocentric chromosome pair, or in some cases slightly displaced toward the interstitial region of the short arm, as previously observed in *Ac. balzani* (Barros et al. 2024). This conserved pattern within *Acromyrmex* contrasts with that of other leaf-cutting ants. In *Atta* species, the 45S rDNA is consistently positioned in the pericentromeric region of the fourth metacentric pair, while in *Amoimyrmex*, the locus is found interstitially on the second metacentric pair (Teixeira et al. 2017; Micolino et al. 2021). These positional differences suggest genus-specific chromosomal contexts and may reflect distinct histories of chromosomal rearrangements, such as pericentric inversions or centromere repositioning. Furthermore, the repeated localization of rDNA loci to pericentromeric or interstitial regions may be linked to the reduced likelihood of deleterious ectopic recombination events, which are more frequent in terminal regions (Teixeira et al. 2021b). In parallel, recent analyses across Formicidae have documented both conserved and variable rDNA positioning patterns, supporting the idea that such loci can serve as informative markers for tracking chromosomal evolution within and across ant subfamilies (Damasceno et al. 2024). Such genomic compartmentalization may thus contribute to the evolutionary stability of rDNA-bearing chromosomes in some lineages, while allowing positional shifts in others. Taken together, these findings support the view that, although rDNA site number is evolutionarily constrained, its chromosomal positioning is more labile and may evolve under lineage-specific structural pressures (see Cardoso and Cristiano 2021, 2024).

Our study addressed three key questions concerning the chromosomal organization of 18S rDNA loci in leaf-cutting ants. First, we demonstrated that while the number of major rDNA loci is conserved across *Acromyrmex*, *Amoimyrmex*, and *Atta*—with a single locus per haploid genome—their chromosomal positioning exhibits clear lineage-specific patterns: terminal in *Acromyrmex*, interstitial in *Amoimyrmex*, and pericentromeric in *Atta*, corroborating previous studies (Teixeira et al. 2017; Micolino et al. 2019b). This positional variation reflects evolutionary plasticity rather than strict conservation. Second, we found that these localization patterns are phylogenetically structured, with each rDNA configuration restricted to a specific genus, indicating a detectable phylogenetic signal despite limited sampling. Third, by mapping rDNA loci across distinct chromosomal compartments, such as telomeres, centromeres, and interstitial regions, we show that rDNA sites can serve as informative cytogenetic markers for inferring compartmental evolution within fungus-farming ants.

Our findings align with recent insights from a broader phylogenomic and cytogenetic analysis of Hymenoptera (Menezes et al. 2021), which confirmed that a single 45S rDNA locus per haploid genome is likely the ancestral condition for aculeate lineages, including ants. Furthermore, their results revealed a nonrandom, lineage-specific distribution of rDNA sites, shaped in part by chromosomal fissions and preferential positioning in pericentromeric and proximal regions – patterns congruent with the M4-i and M2-i configurations observed here for *Atta* and *Amoimyrmex*, respectively.

These findings emphasize the dual role of rDNA loci as both stable cytogenetic landmarks and dynamic genomic elements that reflect lineage-specific chromosomal evolution. Notably, the positional dynamics of rDNA loci may also have functional implications for nucleolar organization and nuclear architecture. Given that rDNA clusters correspond to nucleolus organizer regions (NORs), their chromosomal positioning could influence nuclear topology, transcriptional regulation, and chromatin compartmentalization. In lineages such as *Amoimyrmex* and *Atta*, where rDNA is consistently located in interstitial or pericentromeric regions, this may reflect a selective advantage by promoting stable nucleolar architecture and reducing the risk of chromosomal instability. Conversely, the terminal positioning observed in *Acromyrmex* may be associated with greater karyotypic plasticity, potentially facilitating chromosomal diversification in this genus. Future investigations integrating chromatin topology, gene expression dynamics, and expanded phylogenetic sampling will be essential to assess whether rDNA repositioning is purely structural or bears adaptive significance.

## Footnotes

The primers used to amplify 18S rDNA in this study are frequently attributed to Pereira (2006), as cited in Lopes et al. (2020), who described them for M. quinquefasciata. However, they are also referred to as “unpublished” in Teixeira et al. (2021b) and were previously mentioned in Lopes’ dissertation (Lopes 2006) without proper attribution. Our investigation suggests that neither Pereira nor Lopes originally designed them. Instead, Cunha (2002) used the same primers in a dissertation on Anacardium Linnaeus, 1753, and this work is cited in Galdino et al. (2010). Despite attempts to contact the authors, the original source remains unclear. We suspect the primers are universal or adapted from earlier sources (e.g., Medina et al. 2001). Sequencing the probe and confirming its molecular characteristics in this study aimed to ensure a validated baseline for rDNA-FISH analyses in Hymenoptera.
